# The link between the sphingolipid rheostat and obstructive sleep apnea

**DOI:** 10.1038/s41598-023-34717-4

**Published:** 2023-05-11

**Authors:** Péter Horváth, Lilla Büdi, Dániel Hammer, Rita Varga, György Losonczy, Ádám Domonkos Tárnoki, Dávid László Tárnoki, Martina Mészáros, András Bikov

**Affiliations:** 1 Department of Pulmonology, Semmelweis University, Tömő utca 25-29, 1083 Budapest, Hungary; 2 Medical Imaging Centre, Semmelweis University, Budapest, Hungary; 3 University Hospital Zürich, Zurich, Switzerland; 4 Manchester University NHS Foundation Trust, Manchester, UK

**Keywords:** Medical research, Biomarkers, Translational research

## Abstract

Chronic inflammation induced by hypoxia during sleep is an important mechanism of microvascular damage in OSA patients. In this study, we investigated the role of the sphingosine rheostat, which has diverse inflammatory effects. Thirty-seven healthy subjects and 31 patients with OSA were recruited. We collected data on demographics and comorbidities. Plasma sphingosine-1-phosphate and ceramide antibody concentrations were measured by ELISA. The results were compared between the OSA and control groups, and the correlations between these measurements and markers of disease severity and comorbidities were explored. Ceramide antibody levels were significantly elevated in OSA patients (892.17 ng/ml) vs. controls (209.55 ng/ml). S1P levels were also significantly higher in patients with OSA (1760.0 pg/ml) than in controls (290.35 pg/ml, p < 0.001). The ceramide antibody concentration showed correlations with BMI (ρ = 0.25, p = 0.04), CRP (ρ = 0.36, p = 0.005), AHI (ρ = 0.43, p < 0.001), ODI (ρ = 0.43, p < 0.001), TST90% (ρ = 0.35, p = 0.004) and the lowest oxygen saturation (ρ =  0.37, p = 0.001) in the whole study population but not when patients with OSA were analyzed separately. The elevated ceramide antibody and sphingosine-1-phosphate concentrations in patients suffering from OSA suggests their involvement in the pathomechanism of OSA and its comorbidities.

## Introduction

Obstructive sleep apnea (OSA) is a disorder that is defined by the repeated collapse of the pharyngeal airways during sleep. It is a risk factor for the development of cardiovascular disease, with vascular inflammation induced by chronic intermittent hypoxemia playing a part^1^. However, despite extensive research, the elements of vascular inflammation in OSA are not fully defined. Identification of these processes may reveal potential treatable traits, as unfortunately, the current gold standard treatment for OSA, that is, continuous positive airway pressure (CPAP) therapy, has not been proven to reduce cardiovascular risk^2^.

Sphingolipids are lipid mediators that play a critical role in cellular membrane formation, cell proliferation, and apoptosis and have autocrine roles in regulating inflammation and coagulation^3,4^. In line with this, they are involved in various autoimmune, cardiovascular and metabolic diseases as well as malignancies^5–10^.

Ceramides have a central role in the metabolism of sphingolipids. Their production is induced by proinflammatory cytokines, such as tumor necrosis factor-α (TNF-α) or interleukin 1β^3^, which are both increased in OSA^11^. Once released, ceramides are bound to low-density lipoprotein (LDL) particles and are involved in the development of insulin resistance and the promotion of inflammation^12^. Ceramides might play a role in endothelial dysfunction by inducing apoptosis in endothelial cells in response to stress stimuli^13^. In radiation-induced inflammation, anti-ceramide antibodies have a protective role against inflammation^14^. There is also evidence that antibodies against gangliosides, another group of sphingolipids, play a role in nerve damage in a variety of neurological disorders (e.g., multiple sclerosis and peripheral nerve damage)^15–18^. Anti-ceramide antibodies can disrupt and alter the ceramide signaling pathway. Along these lines, monoclonal antibodies targeting sphingolipid signaling pathways have been intensively studied for the treatment of cancer^19^. Ceramides can further be metabolized to sphingosine 1-phosphate (S1P), which is another important molecule involved in inflammation^3^. Similar to ceramides, the production of S1P is induced by TNF-α in addition to thrombin and growth factors, such as vascular endothelial growth factor (VEGF)^4^. Both thrombin^20^ and VEGF^21^ are elevated in OSA, pointing toward the theoretically increased production of S1P. Following secretion, S1P is mainly bound to apolipoprotein M on high-density lipoprotein (HDL) particles^22^. S1P has a dual role in atherosclerosis. On the one hand, it contributes to vasodilation by activating endothelial nitric oxide synthase^23^. On the other hand, it induces the production of chemokine (C–C motif) ligand 20, which is a potent lymphokine^24^, and potentiates the release of procoagulant and adhesive molecules from platelets, such as P-selectin or von Willebrand factor^4^. The latter two molecules were shown to be increased in OSA^25,26^.

Despite the aforementioned evidence that ceramides and S1P may play a role in OSA, they have not been investigated before. Therefore, the aim of our study was to analyze these molecules in subjects with and without OSA. We also correlated their concentrations to markers of disease severity and associated comorbidities.

## Materials and methods

### Subjects and design

Sixty-eight participants were recruited from those patients who were referred to the Sleep Unit of the Department of Pulmonology, Semmelweis University, due to suspected OSA (i.e., snoring, witnessed apneas, daytime somnolence and/or comorbidities). None of the patients had previously been diagnosed with OSA, nor had they been treated with CPAP or mandibular advancement devices. Exclusion criteria included any uncontrolled chronic disease, history of any malignancy within 10 years, and infection within 2 months. Data for screen failures were not captured.

In the evening, after participants had filled out the Epworth Sleepiness Scale (ESS) and their medical history was recorded, venous blood was collected into EDTA tubes for biomarker measurement. This was followed by an attended full-night polysomnography or cardiorespiratory polygraphy.

The study was approved by the local Ethics Committee (Semmelweis University, TUKEB 30/2014 and RKEB 172/2018), and informed consent was obtained from all participating volunteers. All measurements were performed in accordance with the relevant guidelines and regulations.

### ELISA measurements

EDTA-treated blood samples were centrifuged within 2 h at 1500 RPM for 10 min at 4 °C. Immediately following centrifugation, plasma was separated into 250-µL aliquots, which were stored at − 80 °C until analysis. Samples were thawed just before the ELISA measurements. The MBS3804520 Human Ceramide Antibody ELISA Kit and MBS2516132 Human S1P (sphingosine-1-phosphate) ELISA Kit were used to determine the plasma levels of ceramide antibody and sphingosine-1-phosphate, respectively (MyBioSource, San Diego, California, USA). We followed the manufacturer’s manual to measure these biomarkers; the samples were diluted eight-fold before measurements. Blood samples were measured in duplicate, and the intra-assay variation coefficients were 8.6% for the ceramide antibody assay and 4.2% for the sphingosine-1-phosphate assay.

### Sleep studies

Inpatient overnight polysomnography (n = 41) and cardiorespiratory polygraphy (n = 27) were performed using Somnoscreen Plus Tele PSG and RC devices (Somnomedics GMBH Germany). Sleep stages, movements and cardiopulmonary events were scored manually according to the American Academy of Sleep Medicine (AASM) guidelines^27^. The total sleep time (TST), sleep period time (SPT) and minimum oxygen saturation (MinSatO_2_) were recorded. Apnea was defined as at least 90% nasal airflow reduction lasting for at least 10 s, while hypopnea was defined as at least 30% nasal airflow reduction lasting for at least 10 s that was associated with at least 3% desaturation (for both polysomnography and polygraphy) or arousal (for polysomnography). The apnea–hypopnea index (AHI), oxygen desaturation index (ODI), and percentage of total sleep time spent with oxygen saturation below 90% (TST90%) were calculated. An AHI ≥ 5/hour was diagnostic for OSA. The patient group was divided into the mild (AHI 5–14.9/hour), moderate (AHI 15–29.9/hour) and severe subgroups (AHI ≥ 30/hour).

### Statistical analysis

The Shapiro‒Wilks test was used to test and assess normality, which showed a nonparametric distribution for ceramide antibody and S1P levels. The Mann‒Whitney U test and chi-square test were used to compare clinical and demographic characteristics as well as biomarker levels between the OSA and control groups. We applied a nonparametric analysis of covariance (ANCOVA) test adjusted for age, sex and body mass index (BMI) to investigate the differences in biomarker levels between patients and controls. Nonparametric ANCOVA, adjusted for age, sex and BMI, was also applied to investigate the relationship between biomarkers and disease severity stratified by the AHI followed by Tukey’s post hoc test. Plasma biomarker levels were correlated with clinical variables by using Spearman’s test and logistic regression analysis. Statistical analyses were carried out with JASP 0.14.1 (University of Amsterdam, Amsterdam, The Netherlands) and R v. 4.1.3 (R Statistical Foundation, Vienna, Austria). Graphs were plotted with the ggplot2 package for R. p values < 0.05 were considered significant. Data are presented as the mean ± SD or the median and range. The sample size was estimated to detect differences of at least 70% of the standard deviation (0.70 effect size) in either ceramide antibody or S1P levels between the two groups with a power of 0.80 and α error probability of 0.05^28^.

## Results

### Demographic characteristics

A summary of the descriptive statistics can be found in Table 1. The subjects with OSA were older and had a higher BMI. There were more subjects with hypertension among the patients. There was no significant difference in the distribution of diabetes mellitus type 2 or cardiovascular disease. Patients with OSA had significantly higher CRP, triglyceride, AHI, and oxygen desaturation index (ODI) levels and significantly lower HDL-C levels; however, there was no difference between the Epworth Sleepiness Scale scores.

**Table 1:** Clinical characteristics of the study population.

	Control N = 37	OSA N = 31	p value
Age (years)	47/20–74	60/34–69	< 0.001
Sex (males%)	32	45	0.40
BMI (kg/m^2^)	24.34/17.21–41.01	31.22/20.82–47.86	< 0.001
Hypertension (%)	41	68	0.04
Diabetes (%)	5	19	0.16
Cardiovascular disease (%)	5	16	0.15
Epworth Sleepiness Scale	6.21 ± 3.74	5.68 ± 3.17	0.57
Total cholesterol (mmol/l)	5.34 ± 0.97	5.23 ± 1.19	0.79
HDL-C (mmol/l)	1.87 ± 0.58	1.45 ± 0.81	< 0.001
LDL-C (mmol/l)	2.99 ± 0.94	3.07 ± 0.97	0.91
Triglycerides (mmol/l)	1.1 ± 0.42	1.81 ± 0.84	< 0.001
CRP (mg/l)	1.12/0.05–5.59	3.95/0.14–45.9	< 0.001
AHI (1/h)	2.3/0.0–4.8	20.4/6.8–106.7	< 0.001
ODI (1/h)	0.8/0.0–4.0	19.4/2.1–105.2	< 0.001
TST (min)	404.5/278.0–486.5	409.25/101.5–504.0	0.58
SPT (min)	426.5/288.0–538.0	435.5/109.5–519.0	0.87
MinSatO_2_ (%)	91/87–95	84/52–94	< 0.001
TST90%	0.0/0.0–1.0	3.6/0.0–75.1	< 0.001
Ceramide Ab (ng/ml)	209.55/36.02–1725.29	892.17/4.02–1494.21	< 0.001
S1P (pg/ml)	290.35/41.68–1760.0	1760.0/99.13–1760.0	< 0.001

Data are presented as percentages or the mean ± standard deviation for parametric variables and as the median and interquartile range for nonparametric variables.

HDL-C, high-density lipoprotein cholesterol; LDL-C, low-density lipoprotein cholesterol; CRP, C-reactive protein; AHI, apnea–hypopnea index; ODI, oxygen desaturation index; TST, total sleep time; SPT, sleep period time; MinSatO_2_, minimum oxygen saturation; TST90%, total sleep time with saturation under 90%; Ceramide Ab, ceramide antibody; S1P, sphingosine-1-phosphate.

### Ceramide antibody levels

**Figure 1:**
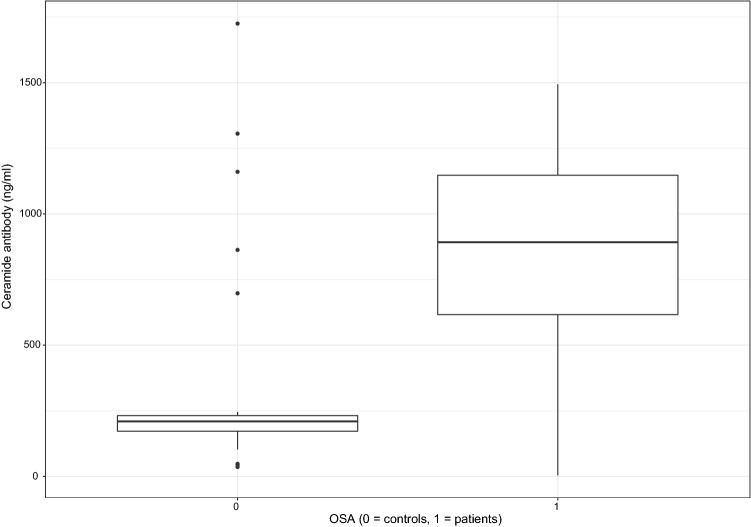
The ceramide antibody concentrations were significantly higher (p < 0.001) in OSA patients.

Ceramide antibody levels were 209.55 ng/ml (36.02–1725.29) vs. 892.17 ng/ml (4.02–1494.21) in controls and OSA patients, respectively (p < 0.001, Fig. 1). The difference remained significant following the adjustment for age, sex, and BMI (p < 0.001).

### Correlation between clinical variables and ceramide antibody levels

We found a significant correlation between the ceramide antibody concentrations and clinical variables in the whole study population. There was a direct correlation with BMI (ρ = 0.25, p = 0.04), CRP (ρ = 0.36, p = 0.005), AHI (ρ = 0.43, p < 0.001), ODI (ρ = 0.43, p < 0.001) and TST90% (ρ = 0.35, p = 0.004). On the other hand, a significant inverse correlation was identified between ceramide antibody levels and the lowest levels of oxygen saturation (ρ = − 0.37, p = 0.001). Ceramide antibody concentrations proved to be independent of sex (p = 0.70) and the presence of hypertension (p = 0.36), diabetes mellitus (p = 0.79) and cardiovascular disease (p = 0.16). Ceramide antibody levels did not correlate with age, lipid values or the ESS (all p > 0.05).

**Figure 2:**
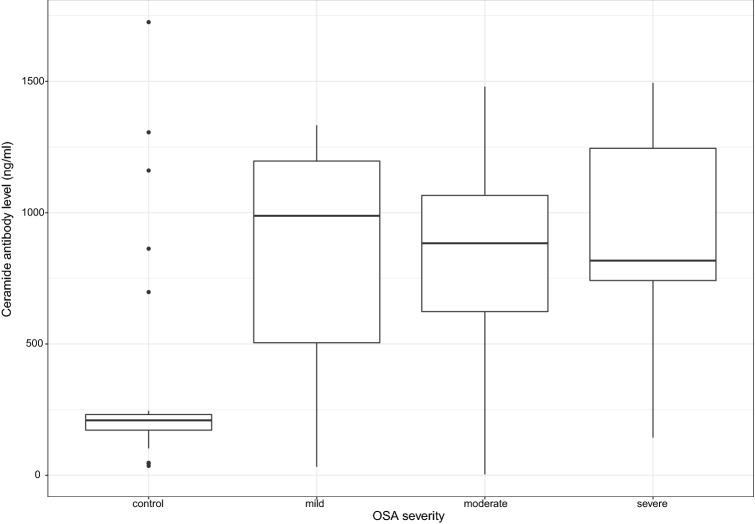
There was a moderate positive correlation between the ceramide antibody concentration and the apnea–hypopnea index (ρ = 0.43, p < 0.001).

When dividing patients into mild (n = 8), moderate (n = 14) and severe (n = 9) subgroups, ceramide antibody levels were in each OSA severity subgroup compared to controls (p = 0.02, p = 0.02, p = 0.008 for mild, moderate and severe subgroups, respectively, Fig. 2); however, there was no difference between any of the OSA subgroups. In line with this, analyzing patients with OSA separately, there was no correlation between ceramide antibody levels and markers of disease severity.

### Sphingosine-1-phosphate levels

**Figure 3:**
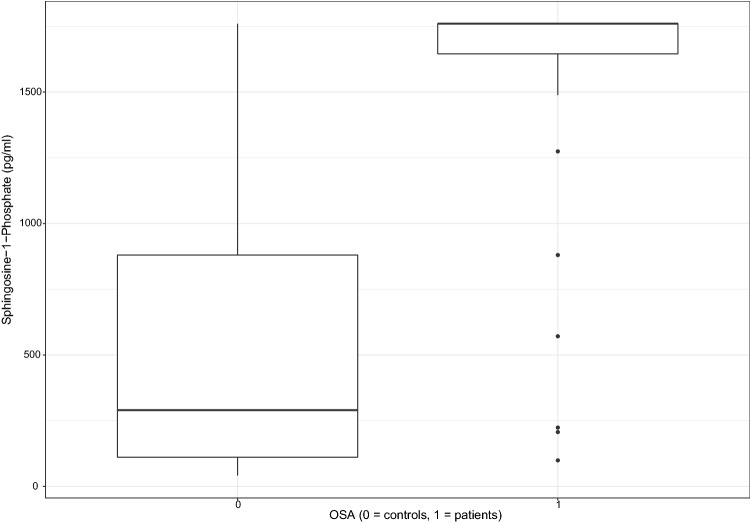
OSA patients had significantly higher sphingosine-1-phosphate concentrations (p < 0.001); however, most values in the disease group were over the limit of detection of the ELISA.

S1P levels were significantly higher in patients with OSA (1760.0 pg/ml, 99.13–1760.0) than in controls (290.35 pg/ml, 41.68–1760.0, p < 0.001). This difference was still significant following adjustment for age, sex, and BMI (p < 0.001). However, most patients had an S1P level above the upper limit of the ELISA kit. These values were treated as the upper limit value. Therefore, we did not proceed with further subgroup or correlation analyses. The graphical representation of S1P values can be seen in Fig. 3.

## Discussion

We found that the levels of S1P and ceramide antibodies are elevated in patients with OSA, suggesting that sphingolipids are involved in the pathomechanism of OSA.

Ceramide antibodies are already upregulated in patients with mild OSA disease. Due to the cross-sectional nature of the study, the reasons for the increase can only be hypothesized. OSA is associated with systemic inflammation^11^, which can induce ceramide synthesis^3^. In line with this, C-reactive protein levels were increased in patients with OSA and correlated with ceramide antibody concentrations. Anti-sphingolipid antibodies were shown to exhibit a T-cell-independent inflammatory response^15^. In addition, ceramide production is directly augmented by hypoxemia^29^, which could be the reason for the direct correlation between ceramide antibody concentrations and markers of overnight hypoxemia. We also found a significant correlation between BMI and ceramide antibody levels. There is a strong relationship between obesity and ceramide metabolism, as white adipose tissue contains large amounts of sphingolipids^30^. However, the relationship between ceramide antibodies and the AHI was stronger than that with BMI. Moreover, differences between the OSA and control groups were present following adjustment for BMI. These results suggest that increased ceramide antibody levels in OSA were not due to obesity; however, this needs to be investigated in certain interventional experimental settings (i.e., changes in ceramide antibody levels either following weight loss or CPAP). Ceramides are also involved in the regulation of body weight and energy homeostasis^31^. It is therefore possible that the elevated ceramide antibody levels are the cause rather than the consequence of high body weight. Nevertheless, the relationship between BMI and ceramides could be essential in OSA, as it has recently been shown that OSA-associated cardiovascular risk is significantly driven by obesity^32^. Ceramides are proapoptotic molecules^33^ and could potentially contribute to the increased apoptotic potential in OSA^34^. In particular, they promote the downregulation of survivin^33^, which is consistent with the decreased survivin concentrations in OSA^35^. Interestingly, ceramides also increase the production of the anti-inflammatory clusterin^36^, which was reported to be elevated in OSA^37^. In addition, ceramides play a role in hypoxia-related vasoconstriction^38^. Elevated ceramide levels in plasma membranes will create rafts that have certain receptor configurations^39,40^. The binding of antibodies to these ceramide-rich rafts could change the configuration of plasma membrane rafts and counteract the effect of ceramide accumulation. Antibodies against ceramide might have a protective effect against hypoxia-induced inflammation, as these antibodies were described to be effective against radiation-induced gut inflammation and leprosy-associated nerve damage^14,18^. This is in accordance with our results, which suggest that ceramide antibody levels are elevated in OSA and are correlated with markers of disease severity. To counteract increased ceramide signaling, whether this elevation is a passive consequence of elevated ceramide production or a negative feedback mechanism needs to be investigated.

A potential consequence of increased ceramide levels is the increased production of S1P. Ceramides are metabolized into sphingosine and subsequently into S1P. This route is facilitated by ceramidase and sphingosine kinases^4^. Sphingosine kinases are activated by growth factors, insulin and TNF-α^25^, which could be involved in OSA^11,21^. Several cell types can release S1P into the circulation, including immune cells, endothelial cells, fibroblasts, and erythrocytes, but it seems that most of the plasma S1P is released during platelet activation^4^. Therefore, high S1P levels could be due to accelerated coagulation and platelet activation in OSA^41^. The fact that most of the S1P concentrations were above the upper limit of detection in OSA prevented us from performing further exploratory analyses. However, high S1P levels could potentially explain some OSA-related changes. For instance, S1P stimulates the secretion of cortisol^42^ and aldosterone^43^, both of which are elevated in OSA^44,45^ and thus contribute to hypertension and insulin resistance. In addition, sphingosine-1-phosphate has a diverse role in the inflammatory response, such as facilitating the extravasation of lymphocytes from lymphatic tissue^46^, inducing inflammation through CXCL-10 and CCL-5^47^ and altering a cell’s gene expression by modulating histone-deacetylases^48^. Sphingosine-1-phosphate is associated with cardiovascular disease^49^ and inflammation caused by intermittent hypoxia^50^. However, our results warrant further investigation into these hypotheses, as S1P seems to be a promising protective biomarker in hypoxia-induced inflammation in OSA and cardiovascular comorbidities. Elevated S1P maintains endothelial barrier function and could be a mechanism to counteract inflammation of the microvessels, which is known to be associated with OSA^7^. However, the angiogenic effects of S1P are (at least partially) mediated through low-density lipoprotein receptor-related protein 1^51^, which was found to be decreased in OSA^52^. Therefore, it is possible that the pathogenic roles of S1P overwhelm the protective functions.

Our study has some limitations. First, although it was powered to detect differences in either ceramide antibody or S1P levels between the two groups, the sample size might be too small to draw conclusions on correlations and subgroup differences. Even though the number of participants was determined based on the power calculation, the number of subjects is low, which adds to the limitations of the overall study. Therefore, our conclusions discussed above need to be tested by further studies. Second, the study had a cross-sectional design, and the causality between OSA and sphingolipid metabolism needs to be confirmed by interventional studies with CPAP. We believe our results will serve as the basis to design such studies. Third, previous evidence suggests that there are high ceramide levels in adipose tissues^30^. Although OSA was associated with high ceramide antibody concentrations independent of BMI, to better understand the effect of OSA on sphingolipid concentrations, comparisons should be performed in lean (BMI < 25 kg/m^2^) subjects. As only 3 patients with OSA fell into this category, we did not proceed with a further assessment. Finally, we used cardiorespiratory polygraphy instead of polysomnography as a diagnostic test in some individuals. Polygraphy is an accepted diagnostic test; however, it tends to underestimate disease severity, as it does not capture hypopneas associated with arousals and may overestimate the total sleep time^32^. This discrepancy could have potentially led to inaccuracies when investigating the correlations between biomarkers and markers of disease severity.

In conclusion, this study is the first to investigate the potential role of sphingolipid metabolism in OSA and its comorbidities. We have found that both sphingosine-1-phosphate and ceramide antibody levels are elevated in patients with OSA. Further investigation is needed to better understand these findings and to reveal the exact role of sphingolipid metabolism in the pathogenesis of OSA and its associated comorbidities.

## Data Availability

The datasets used and/or analyzed during the current study are available from the corresponding author on reasonable request.
